# Genetic Variation in *Rhipicephalus sanguineus* s.l. Ticks across Arizona

**DOI:** 10.3390/ijerph19074223

**Published:** 2022-04-01

**Authors:** Maureen Brophy, Michael A. Riehle, Nikki Mastrud, Alison Ravenscraft, Johnathan E. Adamson, Kathleen R. Walker

**Affiliations:** 1 Department of Entomology, University of Arizona, Tucson, AZ 85721, USA; mriehle@ag.arizona.edu (M.A.R.); krwalker@email.arizona.edu (K.R.W.); 2 Medical Branch Department of Pathology, University of Texas, Austin, TX 78712, USA; nikki.mastrud.01@gmail.com; 3 Arlington Department of Biology, University of Texas, Austin, TX 78712, USA; alison.ravenscraft@uta.edu (A.R.); johnathan.adamson@uta.edu (J.E.A.)

**Keywords:** ticks, acarology, *Rhipicephalus sanguineus*

## Abstract

*Rhipicephalus sanguineus* s.l. (Latreille, 1806), the brown dog tick, is the most widely distributed tick species in the world. The two dominant lineages, a temperate group and a tropical group, are recognized as important disease vectors for both dogs and humans. The temperate and tropical lineages overlap in range in some regions of the world, including the southwestern United States, where recent outbreaks of Rocky Mountain spotted fever are linked to *R. sanguineus* s.l. While it is unclear to what extent they may differ in their capacity to transmit pathogens, finer-scale resolution of temperate and tropical lineage distribution may provide insight into the ecology of these two tick groups and the epidemiology of *R. sanguineus* s.l.-vectored diseases. Using diagnostic polymerase chain reaction assays, we examined the geospatial trends in *R. sanguineus* s.l. lineages throughout Arizona. We found the temperate and tropical lineages were well delineated, with some overlap in the eastern part of the state. In one county, tropical and temperate ticks were collected on the same dog host, demonstrating that the two lineages are living in sympatry in some instances and may co-feed on the same host.

## 1. Introduction

Ticks (Acari: Ixodida: Ixodidae) are important vectors of many human and animal diseases, especially within North America. Among the most significant of these diseases is Rocky Mountain spotted fever (RMSF), a bacterial infection caused by *Rickettsia rickettsii*, which kills more people within North America than any other tickborne disease [1]. In 2018, the Centers for Disease Control and Prevention (CDC) reported 5544 cases of spotted fever rickettsioses, including RMSF reported in the United States, a twelve-fold increase since the year 2000 [2,3]. Most of the reported cases occurred in southeastern states, including North Carolina, South Carolina, Tennessee, Oklahoma, and Arkansas, where the principal vector is *Dermacentor variabilis*, and Montana, Idaho, and Wyoming, where the principal vector is *D. andersoni*.

*Rhipicephalus sanguineus sensu lato*-vectored RMSF has been recognized in Mexico since the 1930s, though data on estimated incidence is limited [4]. Since the first case of locally transmitted RMSF was identified in Arizona in 2003, there have been nearly 500 cases and 28 deaths on six tribal reservations, with a case fatality rate almost 15 times higher than the national rate [5,6,7]. While the disease is easily treated with antibiotics, it can be fatal if left untreated. Delays in seeking health care and the resemblance of symptoms to many other diseases further exacerbate the mortality rate of RMSF in these communities [6].

Dogs are the preferred hosts of *R. sanguineus* s.l., but the ticks will feed on humans as incidental hosts and can transmit *R. rickettsii* at all life stages. The close relationship between domestic dogs and humans enables infestations of *R. sanguineus* s.l. in and around human homes and allows the tick to complete its entire life cycle indoors [8]. Throughout Arizona and northern Mexico, the warm climate and peridomestic habitat provide suitable conditions for the ticks to remain active year-round [9]. Affected communities report a high incidence of free-roaming dogs, which can spread infected ticks among households [5,6,8]. The association of *R. sanguineus* s.l. with RMSF may have important implications for the epidemiology and expansion of this disease to other parts of the world and should be explored in greater detail.

### Genetic Variation of Rhipicephalus sanguineus s.l.

*R. sanguineus* s.l. refers to a complex of 11–17 related species or subspecies with a worldwide distribution [9,10,11]. The existence of more than one species within this taxon was first recognized by Szabo et al. in 2005 [12]. Historically, a non-specific morphological description and loss of the holotype have led systematists to disagree on how to classify the species complex [13,14]. Morphological distinctions among the two main recognized *R. sanguineus* s.l. lineages (a tropical group and a temperate group) and other *Rhipicephalus* species (specifically *R. turanicus*) are minute, further complicating the taxonomic status [15]. In 2018, Nava et al. [13] designated a neotype and renewed morphological and molecular descriptions of the species and identified the previously designated temperate lineage as *R. sanguineus sensu stricto*. The aforementioned tropical lineage within the Americas is currently under revision, with specimens from Australia and southern Asia declared a separate species, *R. linnaei*, by Šlapeta et al. (2021) [16]. To date, however, there has been no revision that includes the tropical lineage as described in North America. While the redesignations are acknowledged as valid, given the current transitionary state of the taxonomical status of the *R. sanguineus* species complex, ticks will be referred to in terms of temperate and tropical lineage throughout this text.

While it has been suggested that the two main *R. sanguineus* s.l. lineages occupy distinct ecological niches [17,18,19,20,21,22], various studies have found both the temperate and tropical groups living in sympatry in central eastern Arizona [17,22,23]. A recent study by Sanchez-Montes et al. (2021) found both lineages present in northern Mexico, with the tropical lineage widespread throughout the country and the temperate lineage present in Chihuahua and Sonora states [21]. Both lineages may harbor *Rickettsia* spp. bacteria, but to date, only the tropical lineage has been associated with the distribution of RMSF in Mexico [18,19,24]. It is unclear whether *R. sanguineus* s.l. lineages differ in their capacity to transmit *R. rickettsii*. However, various experimental transmission studies suggest that the temperate and tropical lineages do differ in their ability to transmit a variety of other pathogens, including *Ehrlichia canis*, *Hepatozooan canis*, and *Anaplasma platys* [25,26,27,28,29,30]. Understanding the distribution of the two *R. sanguineus* s.l. species complex ticks within Arizona may, therefore, provide insight into the epidemiological distribution of *R. sanguineus* s.l.-vectored diseases across the state in the future.

Much work has been done to investigate the molecular dissimilarities between divergent populations within the *R. sanguineus* species complex. Given the higher rate of base substitution relative to most nuclear genes, mitochondrial DNA sequences are useful phylogenetic markers for clades with relatively recent divergence [31]. A systematic review of molecular markers for acarological phylogenetics by Cruickshank (2002) references the 12S rRNA gene as being historically useful to investigate intraspecific relationships within tick species [32]. The mitochondrial 12S rRNA gene is frequently targeted for molecular identification of *R. sanguineus* s.l. taxa [12,17,23,33], often in conjunction with 16S rRNA [14,16,20,22,23,34,35,36,37,38]. Attempts to verify mitochondrial findings with nuclear genes have thus far proven unsatisfactory. Abdullah et al. (2016) compared 12S, 16S, CO1, ITS2, and 18S sequences of *R. sanguineus* s.l. ticks with those of *Hyalomma dromedarii* [39]. The authors determined that while the mitochondrial genes were suitable for phylogenetic analyses, neither 18S nor ITS2 were suitable for such purpose in tick taxonomy. Indeed, Nava et al. (2018) and Šlapeta et al. (2021) relied upon mitochondrial sequences to delineate species within the *R. sanguineus* complex after finding that nuclear markers, specifically ITS2, were insufficiently variable and therefore not informative or robust enough to provide resolution on the necessary scale [13,16].

The aim of this work was to determine the spatial distribution of *R. sanguineus* s.l. lineages within Arizona. A diagnostic polymerase chain reaction (PCR) protocol was developed using lineage-specific primers to differentiate between the temperate and tropical lineages of *R. sanguineus* s.l. based on the mitochondrial 12S rRNA gene with Sanger sequencing confirmation on a subset of samples. Given the current understanding of the global distribution of *R. sanguineus* s.l. ticks, it was hypothesized that the temperate lineage will be more abundant, particularly in northern latitudes and higher elevations across Arizona. However, the presence of the tropical lineage in the six regions of the state where historic and ongoing transmission of RMSF is occurring was predicted.

## 2. Materials and Methods

### 2.1. Sample Collection

In 2018 and 2019, *R. sanguineus* s.l. ticks of all life stages and engorgement levels were solicited throughout the year as a convenience sample from animal shelters, tribal vector control agencies, and University of Arizona Cooperative Extension offices across Arizona. Most ticks included in this study were collected from dogs at animal shelters (Maricopa, Pima, and Pinal Counties) or in collaboration with local stakeholders during vector control activities, such as RMSF prevention campaigns and mobile rabies clinics. The majority of ticks were collected during peak tick activity, during the months of March through August. Ticks were identified to species complex using standard taxonomic keys [10], and the life stage and sex determined.

### 2.2. DNA Isolation and Sequencing

Ticks were placed in a 1.5 mL tube, submerged in liquid nitrogen, homogenized with a sterile pestle, and treated with 20 mg/mL lysozyme solution (Thermo Fisher Scientific, Waltham, MA, USA). DNA was isolated using the Qiagen QiaAmp DNA Mini Kit, with an AE buffer elution volume of 50 μL.

### 2.3. Primer Design, PCR, and Sequencing

Primers were designed using the NCBI Primer Design Tool and sequences published by René-Martellet et al. [23] (Accession numbers KU255848-56; Supplementary Table S1). Lineage-specific primers for *R. sanguineus* s.l. were based on differences of two bp at the binding sites for both the forward and reverse primers, and amplified a sequence of the mitochondrial 12S rRNA approximately 350–360 base pairs in length (Table 1). The “standard 12S” primer was designed to be inclusive of the lineage-specific primer binding sites and amplified a 400 bp region of the 12S rRNA gene that contains significant nucleotide differences between the lineages.

Positive controls for each lineage were provided by the Centers for Disease Control and Prevention Rickettsial Zoonoses Branch. Each sample was prepared using both temperate and tropical primer pairs within the same thermocycler session (primer sequences and parameters described in Table 1). A 4 μL volume of each reaction was visualized by 1% agarose-gel electrophoresis stained with SYBR Safe (Thermo Fisher Scientific, Waltham, MA, USA). PCR product from 5 samples was cleaned and sequencing confirmed that the lineage-specific assay was correctly amplifying the targeted fragment of 12S rRNA for both lineages.

**Table 1:** Standard and diagnostic 12S rRNA PCR protocol for *R. sanguineus* s.l. lineage detection.

Primers		Reagent	Volume	Thermocycler Steps	
				Cycle	Time
Standard 12S rRNA	Forward: AAACTAGGATTAGATACCCTATTATTTTAG Reverse: CTATGTAAGCACTTA- TCTTAATAAAGAGTG	PCR Water	25-sum of other reagents	Initial denaturation	95 °C for 30 s
		ThermoPol Buffer (New England BioLabs)	2.5	30 cycles:	
		10 mM dNTPs	0.5	Denaturation	95 °C for 15 s
		10 µM FWD Primer	0.5	Annealing	50 °C for 30 s
		10 µM REV Primer	0.5	Extension	68 °C for 60 s
		Taq (New England BioLabs)	0.125	Final extension	68 °C for 60 s
		DNA	3.0	Holding	10°C
Temperate 12S rRNA	Forward: TTTTAGAGGTAAACA- TTGTT Reverse: GCTTAATTCAAATTGA- CATT	PCR Water	10-sum of other reagents	Initial denaturation	95 °C for 30 s
		ThermoPol Buffer (New England BioLabs)	1.0	25 cycles:	
		10 mM dNTPs	0.8	Denaturation	95 °C for 15 s
		10 µM FWD Primer	0.5	Annealing	46 °C for 30 s
		10 µM REV Primer	0.5	Extension	68 °C for 60 s
		Taq (New England BioLabs)	0.1	Final extension	68 °C for 60 s
		DNA	1.0	Holding	10 °C
Tropical 12S rRNA	Forward: TTTTAGAGCTTAACAT- TGTA Reverse: GCTTAATTCAAATTAA- CATC	PCR Water	10-sum of other reagents	Initial denaturation	95 °C for 30 s
		ThermoPol Buffer (New England BioLabs)	1.0	25 cycles:	
		10 mM dNTPs	0.8	Denaturation	95 °C for 15 s
		10 µM FWD Primer	0.5	Annealing	46 °C for 30 s
		10 µM REV Primer	0.5	Extension	68 °C for 60 s
		Taq (New England BioLabs)	0.1	Final extension	68 °C for 60 s
		DNA	1.0	Holding	10 °C

Results were further verified on a subset of specimens from each county via Sanger sequencing using the “standard 12S” primer set. Additional sequencing was performed on a ~425 bp fragment of the 16S rRNA gene to verify results for a subset of specimens [13,36]. 12S rRNA sequencing was performed at the University of Arizona Genetics Core. 16S rRNA sequencing was performed at the University of Texas Arlington. Sequences were manually corrected by visual analysis of the electropherogram using Geneious Prime.

### 2.4. Phylogenetic and Geospatial Analyses

Phylogenies were constructed for both the 12S rRNA and 16S rRNA sequences. Sequences used in this analysis are available on the NCBI Sequence Read Archive. Sequences were aligned using MAFFT [40] in Geneious Prime with the default settings. Maximum-likelihood phylogenetic trees were inferred using RAxML version 8.2.10 [41] employing the GTR + Gamma model of nucleotide substitution. Node support values were calculated using rapid bootstrapping halted at 500 replications. The tree was rooted with the outgroup *R. turanicus*.

Data on lineage was mapped to county level using ArcGIS Pro (ESRI). Presence/absence data were recorded for the two lineages at each sampling site, and elevation and latitude data were inferred based on nearest city/town utilizing the ArcGIS Data and Maps global databases.

## 3. Results

**Figure 1:**
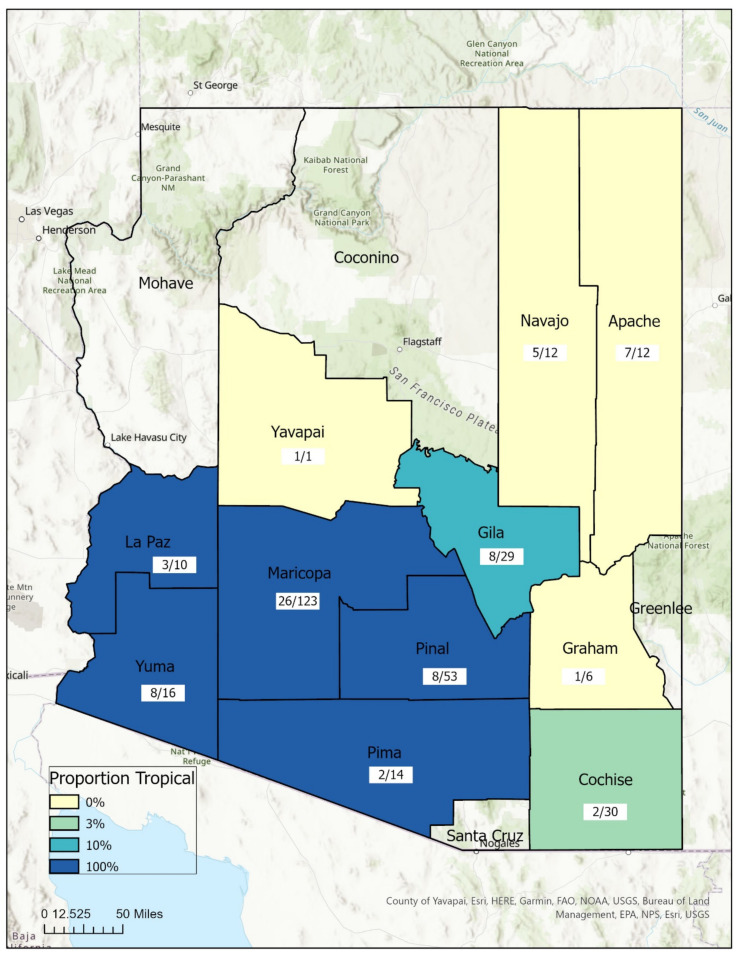
Proportion of *R. sanguineus* s.l. lineages on dog hosts by county. Distribution of temperate and tropical ticks across Arizona. Temperate lineage was exclusively found in yellow counties, tropical in dark blue. Both lineages were found in two counties—Gila and Cochise. Numbers in white boxes are number dogs sampled/number of ticks sampled.

A total of 306 *R. sanguineus* s.l. ticks were included in this study from eleven counties in Arizona (Apache, Cochise, Gila, Graham, La Paz, Maricopa, Navajo, Pima, Pinal, Yavapai, and Yuma, Figure 1). The majority (84.3%) of samples were adults. The bias towards adults likely reflects the difficulty in finding nymphal and larval ticks on hosts, given their size. While most Arizona counties were sampled, many more ticks were sampled in the most populous county, Maricopa (Table 2). Month of collection was recorded for 77.5% of ticks collected. While ticks were collected in every month, with the exceptions of February, October, and December, temperate ticks were only collected from March through July, whereas tropical ticks were collected between August and July. This disparity may be an artifact of the convenience sampling method, as many collaborating stakeholders only hold tick prevention events during peak tick season.

### 3.1. PCR and Sequencing

**Figure 2:**
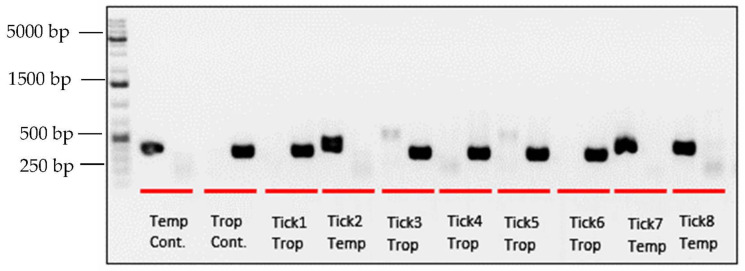
*R. sanguineus* s.l. lineage-specific gel electrophoresis. Red lines delineate reactions using the temperate (left) and tropical (right) primers for each sample. The first sample on the left is a temperate control, with a band from the temperate primer but not the tropical.

As expected, positive controls for the temperate and tropical lineages amplify with the appropriate lineage-specific primer but not the other (Figure 2). Twenty-eight percent (*n* = 86) of the samples amplified using the temperate primers, whereas 72% (*n* = 220) amplified using the tropical primers. Occasionally, there was low amplification with the second lineage-specific primer set, resulting in a faint band in the second lane for a sample (*n* = 39, 14%). This may have been due to non-specific binding, contamination, or an indication of hybridization and paternal leakage of mitochondrial DNA [42]. However, we do not have the data to resolve this. The samples amplified similarly in subsequent repetitions of the assay and we assigned all samples in question to their dominant PCR product.

Sanger sequencing was performed on the 12S PCR amplicon of 117 (38.2%) samples from eleven counties in Arizona and compared against lineage-specific diagnostic PCR results. Four samples (two larvae and two nymphs) were removed from the analysis due to low DNA concentrations and subsequent low-quality Sanger sequencing reads. These samples amplified using the lineage-specific primers but could not be verified with sequencing. For the remaining samples, there was 100% consensus between amplification with lineage-specific primers and sequencing-based identification.

### 3.2. Geospatial Analysis

The distributions of the two *R. sanguineus* s.l. lineages were surprisingly well-delineated across the state. However, both lineages were found in two counties—Gila and Cochise—indicating some overlap in their ranges. Figure 1 depicts the distribution of temperate and tropical ticks and number of dogs sampled across the eleven counties sampled. On two occasions in Gila County, both tropical and temperate ticks were collected on the same dog host, demonstrating that the two lineages are living in sympatry and co-feed on hosts.

**Figure 3:**
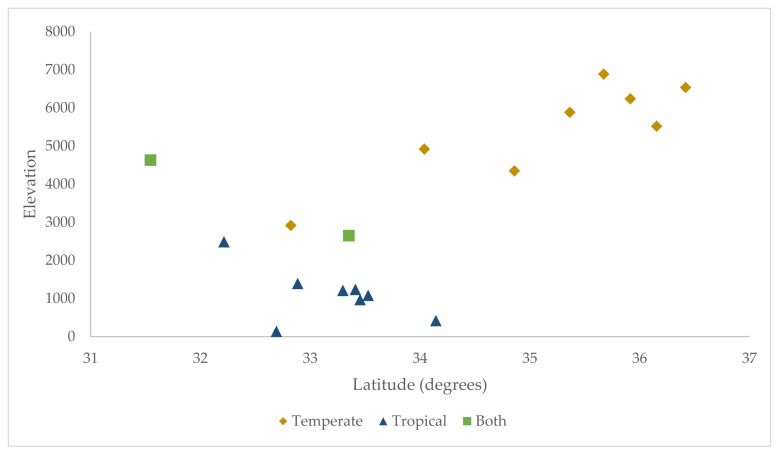
Distribution of *R. sanguineus* s.l. lineages across latitude and elevation of collection sites.

In addition to the sites where both lineages were collected, the temperate and tropical ranges overlapped in latitude. Specifically, both temperate and tropical *R. sanguineus* s.l. ticks were collected between 31.5° and 34° latitude (Figure 3). Interestingly, the temperate lineage was present in higher latitudes at higher elevations, leading the combination of these two variables to near perfectly predict the presence of that lineage (Table 3, Figure 3). However, because latitude and elevation were highly correlated in this dataset, it was not possible to tease apart the effect of the variables within levels of each other without more thorough sampling.

**Table 3:** Distribution of *R. sanguineus* s.l. lineages across elevation (in feet) in Arizona.

Lineage (# Sites)	Mean (Std)	Min	Max
Temperate (10)	5054 (1450)	2650	6888
Tropical (10)	1625 (1316)	141	4633

### 3.3. Phylogenetic Analysis

**Figure 4:**
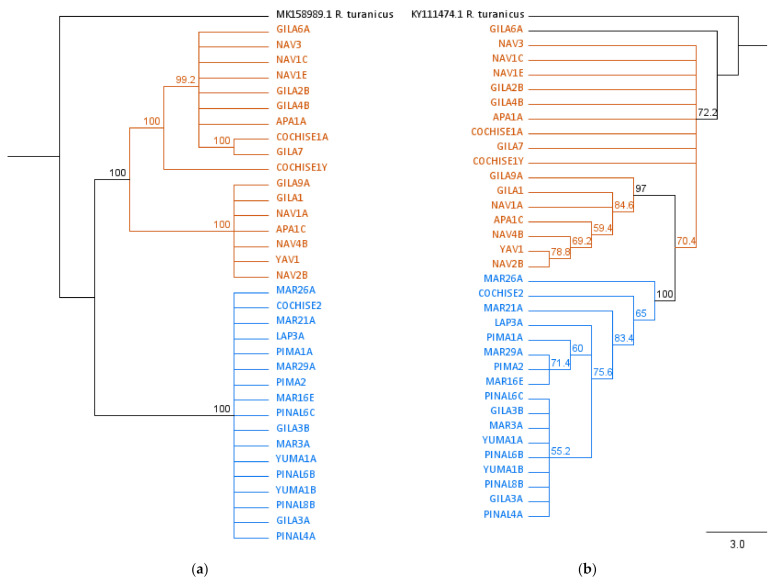
Maximum likelihood phylogenies of *R. sanguineus* s.l. ticks. Maximum likelihood trees based on partial *R. sanguineus* s.l. 12S rRNA (**a**) and 16S rRNA (**b**) sequences, rooted with *R. turanicus* sequences from NCBI GenBank. Ticks identified by diagnostic PCR as temperate lineage in orange, tropical in blue. Numbers at internal nodes represent support values from bootstrap based on 500 replications.

Samples identified as temperate and tropical using diagnostic PCR clustered separately on the 12S rRNA with high bootstrap value (100%) (Figure 4a). On the 16S rRNA phylogeny, all sequences from the tropical lineage formed a distinct clade (Figure 4b). Temperate sequences were basal to the tropical clade but did not form their own distinct clade.

## 4. Discussion

The spatial analysis of *R. sanguineus* s.l. lineage demonstrates that the two lineages overlap in range in Arizona. While this study utilized a convenience sampling method and therefore the lack of a lineage in a county may not be representative of an actual absence, the results support the published literature on the distribution of the temperate lineage [22,34]. We found temperate ticks in the present study in lower latitudes at higher elevations. This matches findings from Sanchez-Montes et al. [21], where the presence of the temperate lineage in northern Mexico was at or above 4000 feet elevation. This also corroborates the interpretation of Zemtsova et al. [22] that the two lineages are climatically restricted within certain temperature ranges. Labruna et al. [43] further elucidated this geospatial relationship by identifying that the temperate lineage has a pronounced diapause in winter months, suggesting adaptation to colder regions, whereas the tropical lineage, which inhabits warmer regions, lacks a distinct diapause.

It is well established that vector-borne diseases, including those transmitted by ticks, increase in incidence as the global climate warms [44]. The results presented here contrasted expectations that the temperate lineage would be dominant in this region based on global patterns of distribution [22,43]. The distribution of the tropical lineage across Arizona may represent an ecological range expansion event northward [19], and suggests potential implications of changing global conditions on a local disease vector. A better understanding of the specific range-limiting factors of the two *R. sanguineus* s.l. lineages is necessary to assess future risks of tickborne diseases vectored by this tick species complex in a warming world.

The diagnostic PCR protocol described here quickly and reliably distinguishes between temperate and tropical lineages of *R. sanguineus* s.l. ticks without the need for additional sequencing. While not sufficient for phylogenetic analyses, it may prove useful for researchers seeking to identify *R. sanguineus* s.l. ticks to lineage without additional investments in time and resources. This protocol may be especially useful in regions where both lineages have been found to screen large numbers of ticks and define the distribution of *R. sanguineus* s.l. with high resolution.

Due to lingering taxonomic questions, several hybridization experiments have been conducted using geographically isolated, allopatric *R. sanguineus* s.l. populations [33,45]. A cross-breeding experiment of two temperate *R. sanguineus* s.l. populations showed reproductive success, and also indicated the occurrence of paternal inheritance of mitochondria and mitochondrial heteroplasmy [45]. Hybridization between tropical and temperate *R. sanguineus* s.l. lineages has also been observed experimentally, although most of the hybrid progeny did not produce viable offspring [33,45]. However, these experiments involved geographically distant tick populations rather than the sympatric tropical and temperate ticks observed in this study. While unresolved in this study, the faint double bands using the lineage-specific primer sets may be an indication of hybridization between the two lineages with paternal mitochondrial DNA leakage. Future researchers may consider investigating the hybridization success of sympatric populations of the temperate and tropical *R. sanguineus* s.l. lineages, and whether an *R. sanguineus* s.l. tick may behaviorally present as one lineage while mitochondrial DNA determines it to be the other. Identification of a nuclear genetic marker with sufficient resolution at this taxonomic level would aid in this line of research.

While *R. sanguineus* s.l. is the primary vector of RMSF in Arizona, much remains to be investigated. The temperate and tropical *R. sanguineus* s.l. lineages are present in Arizona and living in sympatry in some areas. A systematic assessment of the presence/absence of each lineage across the state using non-convenience sampling strategies may highlight additional areas of sympatry. Future research on this topic should focus on whether the two lineages differ in their vectorial capacity for RMSF. Additionally, a more thorough assessment of climatic variables relevant to range limitation and overlap may be helpful to better understand the distribution of the temperate and tropical *R. sanguineus* s.l. ticks in this region of the United States. Finally, researchers should seek evidence of hybridization among sympatric temperate and tropical *R. sanguineus* s.l. lineages to further understand the implications of this on the epidemiology of RMSF and other tickborne diseases.

## 5. Conclusions

This research explores the distribution of the two recognized lineages of *R. sanguineus* s.l., temperate and tropical, at a fine scale in Arizona. We were able to sample ticks from eleven of fifteen counties in the state, including regions of the state affected by *R. sanguineus* s.l.-transmitted RMSF. The data presented here demonstrates a clear pattern of *R. sanguineus* s.l. lineage distribution across both latitude and elevation within Arizona. We also verify the presence of both lineages in two counties in the eastern part of the state. Our findings are somewhat limited by convenience sampling and limited access to dog hosts in some counties, as it is possible that our dog sample sizes in some areas were insufficient to obtain a representative sample of lineage.

## Figures and Tables

**Table 2:** Life stage and sex of *R. sanguineus* s.l. ticks collected.

Life Stage	*n* (%)
Larva	4 (1.3)
Nymph	44 (14.4)
Adult	258 (84.3)
Male	144 (55.8)
Female	114 (44.2)

## Data Availability

The data presented in this study are openly available in the NCBI GenBank (accession no. OM985227-OM985391).

## Supplementary Materials

The following supporting information can be downloaded at: https://www.mdpi.com/article/10.3390/ijerph19074223/s1, Table S1: Primer binding sites from René-Martellet et al., 2017.
